# Study and Test of a New Bundle-Structure Riser Stress Monitoring Sensor Based on FBG

**DOI:** 10.3390/s151129648

**Published:** 2015-11-24

**Authors:** Jian Xu, Dexing Yang, Chuan Qin, Yajun Jiang, Leixiang Sheng, Xiangyun Jia, Yang Bai, Xiaohong Shen, Haiyan Wang, Xin Deng, Liangbin Xu, Shiquan Jiang

**Affiliations:** 1 Key Laboratory of Optical Information Technology, School of Science, Northwestern Polytechnical University, 127 West Youyi Road, Xi’an 710072, China; E-Mails: xujian1989@hotmail.com (J.X.); qin_adpnwpu@163.com (C.Q.); yjjiang@nwpu.edu.cn (Y.J.); jiaxiangyun@mail.nwpu.edu.cn (X.J.); by910305@163.com (Y.B.); 2 The Key Laboratory of Space Applied Physics and Chemistry, School of Science, Northwestern Polytechnical University, 127 West Youyi Road, Xi’an 710072, China; 3 Key Lab of Deepwater Engineering CNOOC Research Institute, Room 808, CNOOC Plaza, Taiyanggong South Road 6#, Chaoyang District, Beijing 100028, China; E-Mails: shenglx@cnooc.com.cn (L.S.); xulb@cnooc.com.cn (L.X.); jiangshq@cnooc.com.cn (S.J.); 4 School of Marine Science and Technology, Northwestern Polytechnical University, 127 West Youyi Road, Xi’an 710072, China; E-Mails: xhshen@nwpu.edu.cn (X.S.); hywang@nwpu.edu.cn (H.W.); 5 Zhanjiang Western South China Sea Oil Survey and Design Co., Ltd., Nanyou Engineering Building, Nandiao Road, Zhanjiang 524057, China; E-Mail: dengxin@cnooc.com.cn

**Keywords:** FBG, riser, stress monitoring, bundle-structure, marine test

## Abstract

To meet the requirements of riser safety monitoring in offshore oil fields, a new Fiber Bragg Grating (FBG)-based bundle-structure riser stress monitoring sensor has been developed. In cooperation with many departments, a 49-day marine test in water depths of 1365 m and 1252 m was completed on the “HYSY-981” ocean oil drilling platform. No welding and pasting were used when the sensor was installed on risers. Therefore, the installation is convenient, reliable and harmless to risers. The continuous, reasonable, time-consistent data obtained indicates that the sensor worked normally under water. In all detailed working conditions, the test results show that the sensor can do well in reflecting stresses and bending moments both in and in magnitude. The measured maximum stress is 132.7 MPa, which is below the allowable stress. In drilling and testing conditions, the average riser stress was 86.6 MPa, which is within the range of the China National Offshore Oil Corporation (CNOOC) mechanical simulation results.

## 1. Introduction

In recent years, the rapid development of the offshore oil industry has contributed greatly to the study of marine equipment. It is a big challenge to keep marine equipment in safe conditions, especially in the deep sea. As an important part of the oil platform, risers are exposed to many kinds of complicated forces in complex working conditions such as drilling and pipe-laying. To avoid damage and failure due to excessive stresses or bending moments in risers, real-time stress monitoring of risers is very important.

Various sensors have been developed to monitor riser safety, but most of them can be classified as electronic sensors [1,2,3,4,5,6,7,8]. In the deep sea environment, sensors need better electrical insulation capability, anti-pressure capability, anticorrosion capability, and less transmission loss. Fiber optic sensors meet these requirements and have been successfully applied in offshore oil fields [9,10,11,12,13]. As early as 2004, Brower *et al.* monitored subsea pipelines and facilities using fiber optic sensors [14]. In 2006, Morrison *et al.* developed a fiber Bragg grating (FBG) sensor with a four-point structure to monitor stresses of steel catenary risers [15]. Then, four-point structure FBG sensors were used to monitor vortex-induced vibrations (VIV) in laboratory riser models [16,17]. Morikawa *et al.* [18] and Weppenaar *et al.* [19] used FBG sensors on flexible pipelines to monitor fracture failures in 2008 and 2014, respectively. Recently in 2015, Vishwesh *et al.* researched failure mechanisms of aluminum tubes under high pressure by using FBG sensors [20].

However, in actual working conditions, the positions of riser stress monitoring depend on the water depth and object requirements. Sensors are hard to move if they are installed in traditional ways which involve pasting or welding. Surface-breaking installations are also harmful to riser corrosion resistance. A riser stress monitoring sensor based on polymer-packaged FBGs was developed to meet these requirements [21], but many disadvantages such as chirps and creeps were found in subsequent long-term laboratory tests. 

In this paper, after applying an all-metal packaging technology [22], a new bundle-structure sensor for riser stress monitoring was developed to overcome these problems. In cooperation with several departments of the China National Offshore Oil Corporation (CNOOC), a marine test of the sensor was completed on the “hysy-981” ocean oil drilling platform in a sea area of south china sea named Lingshui, which is near the city of Sanya. The water depths of the well heads were 1365 m and 1252 m. The test lasted for 49 days and reasonable data was obtained.

## 2. Structure and Principle of Sensor

### 2.1. Structure and Composition

**Figure 1:**
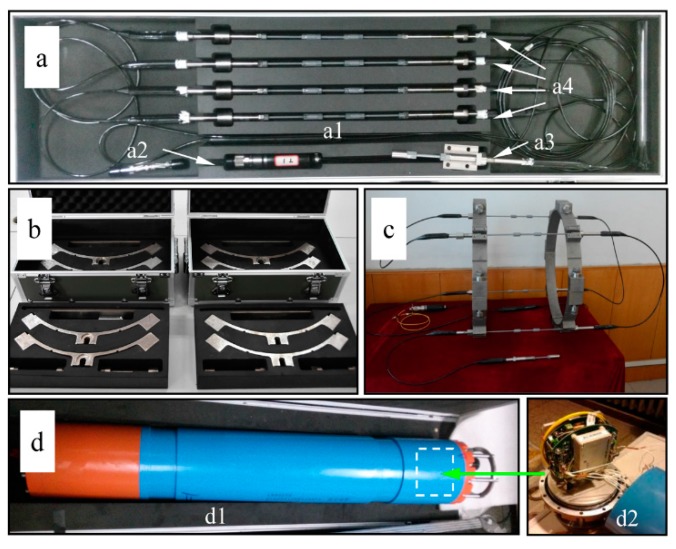
The developed bundle-structure riser stress sensor system. (**a**) A string of five FBG sensors: elastic elements (**a1**), an optical fiber connector (**a2**), a FBG environment-compensating sensor (**a3**), four FBG strain sensors (**a4**); (**b**) two unassembled ring-shaped ties; (**c**) the assembly of five FBG sensors and two ring-shaped ties; (**d**) housing (**d1**) and demodulation module (**d2**).

The riser stress monitoring sensor system consists of two ring-shaped ties, an interrogation module and a string of five FBG sensors, as shown in Figure 1. The five FBG sensors shown in Figure 1a are composed of four FBG strain sensors and a FBG environment-compensating sensor. Elastic elements in the middle of FBG sensors are key components to get the wavelength shifts of FBGs caused by stresses in the risers at the installation site. Bare FBGs in elastic elements are protected by all-metal packages that overcome the chirps and creeps that existed in our previously developed sensors [21]. 

Two ring-shaped ties shown in Figure 1b are disassembled into eight quarter-circle-shaped blocks and eight groups of bolts and nuts. Each block has a counter bore to fix one end of a FBG strain sensor. Figure 1c shows the combination of five FBG sensors and two ring-shaped ties. Four FBG strain sensors are fixed on a bundle structure made by ties and the FBG environment-compensating sensor is single-side fixed. The space between two ties is 440 mm. The longest length of the manufactured elastic elements is 100 mm. Therefore, each FBG strain sensor shown in Figure 1(a4) adds two thinner grey extension tubes to increase the length. 

The interrogation module is put in an anti-pressure housing shown in Figure 1d. It interrogates FBG wavelengths reflected by FBG sensors and transmits these signals to the communication module. Then, the communication module in the housing uploads these signals to the oil platform by sonar [23]. Several processing technologies are used to protect the sensor system from the deep sea environment. Anticorrosive paint is utilized to isolate the elastic elements from sea water. Therefore, seawater corrosion and electrochemical corrosion can be effectively avoided. High water-pressure resistant vulcanizate is employed to protect the connections between submarine cables and the FBG sensor ends. 

### 2.2. Sensing Principle

In order to describe what follows conveniently, the meanings of the parameters involved are defined in Table 1.

**Table 1:** Definition of parameters.

Parameters	Parameter Meanings	Values
*λ*_10_, *λ*_20_, *λ*_30_, *λ*_40_, *λ*_50_	Initial wavelengths of FBG sensors	--
*λ*_1_, *λ*_2_, *λ*_3_, *λ*_40_, *λ*_5_	Measured wavelengths of FBG sensors	--
*P*, *T*	Water pressure change, Temperature change	--
*σ*, *ε*, *M*	Stress, Strain, Bending moment	--
*η*	Sensitivities	--
the subscript “p”, “t”, “z”, “w”	Sensitivity types related to pressure, temperature, tension and bend, respectively	--
the subscript “*x*”, “*y*”	Parameter types related to the *x* axis and the *y* axis in Figure 2	--
the subscript “c”	Parameter type related to strain sensors	--
*I*_z_	Inertia moment of riser, *I*_z_ = *π*(*D*^4^ − *d*^4^)/64	--
*D*	Inner diameter of riser	489 mm
*D*	Outer diameter of riser	533 mm
*D*_c_	Equivalent diameter of the riser stress monitoring sensor	576 mm
*E*	Elastic modulus of riser metal materials	2.05 × 10^11^ Pa
*η*_z_	Tension sensitivities of FBG sensors	1.09 pm/µε

**Figure 2:**
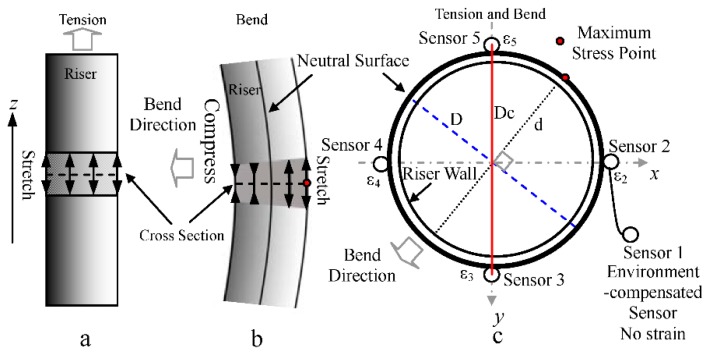
Schematic diagram of the sensing principle. (**a**) Riser in tension; (**b**) Riser under bending; (**c**) Locations of FBG sensors.

As shown in Figure 2, when the riser is in tension, riser tension strains in the same cross-section become larger due to stretching and their values are equal, *ε*_2z_ = *ε*_3z_ = *ε*_4z_ = *ε*_5z_ = *ε*_z_. The riser stress produced by tension is *δ*_z_ = *E*∙*ε*_z_. 

When the riser is bent, the sensors in the bend direction and on the other side are compressed and stretched, respectively [21]. The riser bend strains are symmetrical around the center point of the riser cross-section. Riser bend strains in the locations of Sensor 2 and Sensor 4 are equal and opposite. It is the same for Sensor 3 and Sensor 5. Therefore, *ε*_2w_ = *− ε*_4w_ = *ε_x_*, *ε*_3w_ = *− ε*_5w_ = *ε_y_*, where the *x* direction is from riser cross-section center to Sensor 2, and the *y* direction is from riser cross-section center to Sensor 3. The riser stress produced by a bend in the maximum stress point can be written as:

(1) $$\sigma_{W}=E\sqrt{{\varepsilon_{x}}^{2}+{\varepsilon_{y}}^{2}}$$

(2) $$\sigma_{W}=MY/I_{\text{z}}$$

The relationship between bending moment and bend stress is defined by Equation (2), where *Y* is the distance from the measuring point to the riser neutral surface. At the maximum stress point, *Y* = *D*/2. Therefore, the bending moment is given by:

(3) $$M=2EI_{\text{z}}\sqrt{{\varepsilon_{x}}^{2}+{\varepsilon_{y}}^{2}}/D$$

When the riser is both in tension and bent, its maximum stress becomes:

(4) $$\sigma_{\max}=\sigma_{\text{z}}+\sigma_{\text{w}}$$

Similarly, the strains of a single FBG strain sensor consist of the tension strain *ε*_cz_ and the bending strain *ε*_cw_. The relationships between riser strains and sensor strains are expressed as:

(5) $$\varepsilon =\varepsilon_{\text{z}}+\varepsilon_{\text{w}},\varepsilon_{\text{c}}=\varepsilon_{\text{cz}}+\varepsilon_{\text{cw}},\varepsilon_{\text{z}}=\varepsilon_{\text{cz}},\varepsilon_{\text{w}}=\varepsilon_{\text{cw}}D/D_{\text{c}}$$

In deep sea conditions, the measured wavelength of a single FBG strain sensor is determined by the initial wavelength, the pressure and temperature of water, and the tension and bend of riser. The relationship can be shown as:

(6) $$\lambda =\lambda_{0}+\eta_{\text{p}}P+\eta_{\text{t}}T+\eta_{\text{z}}\varepsilon_{\text{cz}}+\eta_{\text{w}}\varepsilon_{\text{cw}}$$

By mechanics analysis, the relationship between the bend strain sensitivity *η*_w_ and the tension strain sensitivity *η*_z_ is *η*_w_ = *η*_z_∙*D*_c_*/D*. Therefore, we obtain:

(7) $$\lambda =\lambda_{0}+\eta_{\text{p}}P+\eta_{\text{t}}T+\eta_{\text{z}}\left(\varepsilon_{\text{z}}+\varepsilon_{\text{w}}D_{c}^{2}/D^{2}\right)$$

(8) $$\eta_{\text{1p}}=\eta_{\text{2p}}={\eta_{\text{3}}}_{\text{p}}={\eta_{4}}_{\text{p}}={\eta_{5}}_{\text{p}}=\eta_{\text{p}},\eta_{\text{1t}}=\eta_{\text{2t}}={\eta_{\text{3}}}_{\text{t}}={\eta_{\text{4}}}_{\text{t}}={\eta_{\text{5}}}_{\text{t}}=\eta_{\text{t}},\eta_{\text{2z}}={\eta_{\text{3}}}_{\text{z}}={\eta_{\text{4}}}_{\text{z}}={\eta_{\text{5}}}_{\text{z}}=\eta_{\text{z}}$$

(9) $$\left\{ \begin{array}{l} \lambda_{1}=\lambda_{10}+\eta_{\text{p}}P+\eta_{\text{t}}T\\ \lambda_{2}=\lambda_{20}+\eta_{\text{p}}P+\eta_{\text{t}}T+\eta_{\text{z}}\left(\varepsilon_{\text{z}}+\varepsilon_{x}D_{c}^{2}/D^{2}\right)\\ \lambda_{3}=\lambda_{30}+\eta_{\text{p}}P+\eta_{\text{t}}T+\eta_{\text{z}}\left(\varepsilon_{\text{z}}+\varepsilon_{y}D_{c}^{2}/D^{2}\right)\\ \lambda_{4}=\lambda_{40}+\eta_{\text{p}}P+\eta_{\text{t}}T+\eta_{\text{z}}\left(\varepsilon_{\text{z}}-\varepsilon_{x}D_{c}^{2}/D^{2}\right)\\ \lambda_{5}=\lambda_{50}+\eta_{\text{p}}P+\eta_{\text{t}}T+\eta_{\text{z}}\left(\varepsilon_{\text{z}}-\varepsilon_{y}D_{c}^{2}/D^{2}\right) \end{array}\right.$$

As shown in Equation (8), the sensitivities of pressure and temperature of all FBG sensors are equal owing to the use of the same design and the same processing technology. It is the same for the tension sensitivities of the four strain sensors. The dependences of the environmental parameters and the riser strains on the wavelengths of FBG sensors can be described using Equation (9). No strain exists in Sensor 1 because of its single-side fixation. Regarding two environment parts (*η*_p_*P*, *η*_t_*T*) as an independent one, there are only four unknown quantities (*ε_x_*, *ε_y_*, *ε*_z_, *η*_p_*P* + *η*_t_*T*) in Equation (9). From Equation (9), we get:

(10) $$\left\{ \begin{array}{l} \varepsilon_{\text{z}}=\left[\left(\lambda_{2}-\lambda_{20}\right)+\left(\lambda_{3}-\lambda_{30}\right)+\left(\lambda_{4}-\lambda_{40}\right)+\left(\lambda_{5}-\lambda_{50}\right)-4\left(\lambda_{1}-\lambda_{10}\right)\right]/4\eta_{\text{z}}\\ \varepsilon_{x}=\left[\left(\lambda_{2}-\lambda_{20}\right)-\left(\lambda_{4}-\lambda_{40}\right)\right]D^{2}/2\eta_{\text{z}}D_{c}^{2}\\ \varepsilon_{y}=\left[\left(\lambda_{3}-\lambda_{30}\right)-\left(\lambda_{5}-\lambda_{50}\right)\right]D^{2}/2\eta_{\text{z}}D_{c}^{2} \end{array}\right.$$

Finally, the values of bending moment and maximum stress in riser can be obtained easily by substituting Equation (10) into Equations (3) and (4).

## 3. Test Process 

### 3.1. Sensor Installation 

**Figure 3:**
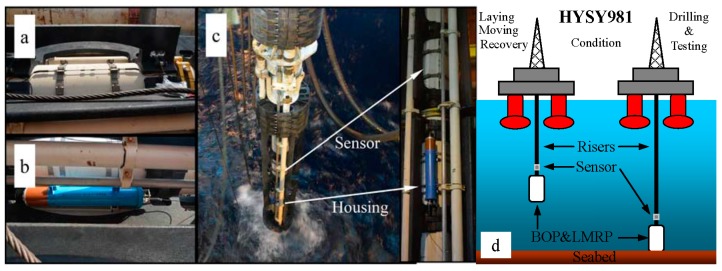
The sensor system assembled on a riser. (**a**) Assembled connected sensors and ties; (**b**) assembled housing; (**c**) close-up of the sensor system lowered into water; (**d**) working conditions schematic of the ocean oil drilling platform “HYSY981”.

The riser stress monitoring sensor system was tested in the ocean oil drilling platform named “HYSY981”. The installation included six steps: determining the mounting positions, mounting ring-shaped ties, mounting the sensors, detecting signals, connecting the fiber optic connectors and providing power. Then, all parts of the sensor system were assembled in the middle of a riser as shown in Figure 3a,b. Figure 3c shows a close-up of the sensor system before it was lowered into the water. From this moment on, the sensor system began to monitor riser stresses and send five FBG wavelengths per hour. 

### 3.2. Riser Working Conditions 

In the sea area named Lingshui in the South China Sea, two oil wells marked as LS17-2-7 and LS17-2-8 were drilled with water depths of 1365 m and 1252 m, respectively. In the 49-day marine test from 14 October to 1 December in 2014, the risers experienced four basic working conditions presented in Figure 3d. Under laying conditions, risers are connected into a string and laid in the water. In this period, the temperature decreases and the water pressure increases as the sensor system depth in the water increases. Moving conditions means that the oil drilling platform is moved from one well to the other. Recovery conditions means that the risers are disconnected and returned to the platform. In the recovery period, the temperature became higher and the water pressure gradually became lower. In drilling and testing conditions, the bottom of riser is connected to the seabed when an oil well is drilled or tested.

## 4. Data and Analysis

**Figure 4:**
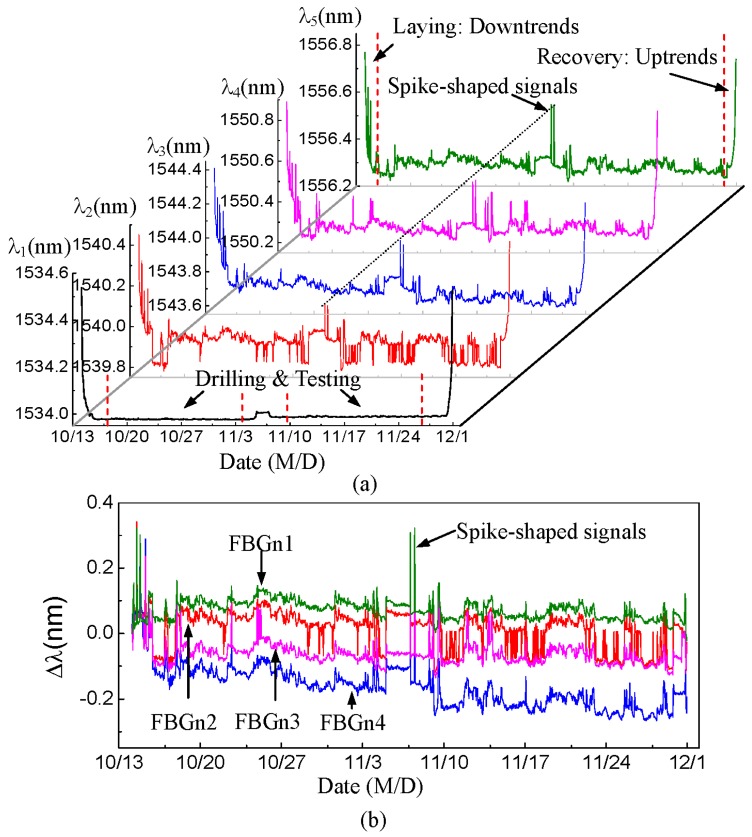
Marine test data of the riser stress monitoring sensor system. (**a**) Measured wavelengths of five FBG sensors; (**b**) compensated data of four FBG strain sensors.

Figure 4a presents the measured wavelengths of the five FBG sensors in the marine test. The abscissa is the time axis and the ordinate represents the measured wavelength values. The signal continuity of the five wavelength curves shows that the sensor system worked well, without sensor damage or signal failure in actual working conditions under water.

The five wavelength curves have downward trends on October 15 when the risers were in laying conditions, and upward trends on 30 November 2014 when risers were in recovery conditions. The previous experimental investigations in the lab [22] indicated that the wavelength values of FBG sensors are proportional to temperature changes and inversely proportional to water pressure changes. According to the trends of temperature and pressure under seawater in riser working conditions, the downtrends and the uptrends of the curves are consistent with the actual shifts of measured wavelengths. In drilling and testing conditions, the risers were connected to the seabed and the temperature and suffered pressure of sensors under deep water can be thought of as constants owing to the given water depth. Moreover, Sensor 1 was not affected by the riser strains, so the stability in the bottom of Curve *λ*_1_ is reasonable.

The sensor data after environmental compensation is shown in Figure 4b, where Δ*λ* = *λ* – *λ*_0_ is the measured wavelength shift of each FBG sensor, and FBGn1 = Δ*λ*_2_ − Δ*λ*_1_, FBGn2 = Δ*λ*_3_ − Δ*λ*_1_, FBGn3 = Δ*λ*_4_ − Δ*λ*_1_, FBGn4 = Δ*λ*_5_ − Δ*λ*_1_. The downtrends and uptrends disappear under laying and recovery conditions because of the practical environmental compensation. In the four compensated curves, some spike-shaped signals appearing at the same time also verify the time consistency of these FBG sensors. The continuity, reasonability and time consistency of the data effectively show that the sensor system had been installed reliably. Vibrations or other environmental conditions had no significant impact on the sensor system in the marine test.

## 5. Results and Discussion

**Figure 5:**
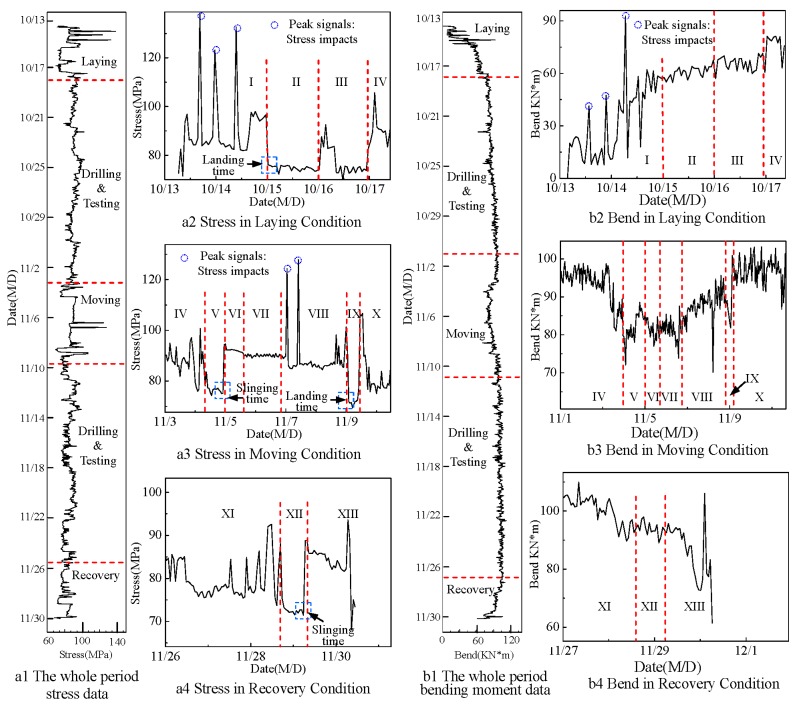
(**a**) Stress results; (**b**) bending moment results.

According to Equations (3) and (4) and the weight (267 tons) of the blowout preventer (BOP) and lower marine riser package (LMRP), the monitored results of stress and bending moment are obtained in Figure 5. Under three basic working conditions, namely laying, moving and recovery, the detailed stress results are shown in Figure 5a(2,3,4), and the detailed bending moment results are shown in Figure 5b(2,3,4), respectively. In each set of basic condition, more detailed conditions are observed.

### 5.1. Stress Analysis

The analysis of stress results are shown in Table 2. In all thirteen detailed periods shown in Figure 5, stress trends can be reasonably explained. This indicates that stress results agree well with the actual work conditions.

**Table 2:** Stress trends under different detailed conditions.

Working Condition		Specific Content	Stress Trends	Main Causes of Changes
Laying	C1	Riser connecting	High and unstable; With some stress impacts;	Risers were influenced by unstable waves, surges and the gravity of BOP and LMRP; sudden braking of the riser crane produced stress impacts.
	C2	BOP pressure test	Low and stable;	There was no BOP and LMRP gravity influence; the platform position was locked right above the well head that straightened the riser string.
	C3	Drilling preparation	High and unstable at the beginning; then stable	Using or changing of tools in drilling caused unstable stresses; risers became stable in testing.
Drilling & Testing	C4	Drilling & Testing	Sometime high and unstable; sometime low and unstable;	Same as Condition C3
Moving	C5	Regain preparation	Low and stable;	Same as Condition C2
	C6	Regain risers	High and unstable;	It is the same as Condition C1 except stress impacts.
	C7	Moving drilling platform	Low and unstable;	When the platform was moved to the second well, seven risers were disconnected to avoid the collision between BOP and the seabed. The depth of sensor was decreased. Remaining risers was influenced by unstable waves, surges and the gravity of BOP and LMRP, but the depth had no change in moving.
	C8	Connecting risers	High and unstable; With some stress impacts;	Same as Condition C1
	C9	BOP pressure test	Low and stable;	Same as Condition C2
	C10	Drilling preparation	High and unstable at the beginning; then stable	Same as Condition C3
Drilling & Testing	C11	Drilling & Testing	Sometime high and unstable; sometime low and unstable;	Same as Condition C3
Recovery	C12	Recovery preparation	Low and stable;	Same as Condition C2
	C13	Recover risers	High and unstable;	Same as Condition C6
Special Times	--	Landing time	much lower in a sudden	BOP and LMRP touched the seabed, no gravity influenced risers.
	--	Slinging time	much higher in a sudden	BOP and LMRP left the seabed, the gravity influenced risers.

In laying conditions, the maximum stress is 132.7 MPa. The yield strength of the riser materials named X80 Steel is 552 MPa and the allowable stress is 220.8 MPa. The maximum stress of the riser measured during the whole monitoring period is below the allowable stress. The initial stress after sensor installation is 78.7 MPa and the final stress before sensor removal is 73.4 MPa. The difference between the values of these two stresses is 5.3 MPa, mainly caused by perpendicularly moving surges forced on the heavy BOP and LMRP. 

### 5.2. Bending Moment Analysis

Seen in Figure 5(b1), the maximum bending moment is less than 106 kNm during the monitoring period. As shown in Figure 5(b3), bending moments decrease under moving conditions when the platform was being moved to the other well. In Figure 5(b4), a large spike-shaped signal appears at the end, indicating that there was a sudden bend impact when the risers were disconnected. The initial bending moment has a big difference from the final one. Considering that BOP and LMRP were influenced by horizontally moving waves, the error is reasonable.

### 5.3. Results Reliability Analysis

**Figure 6:**
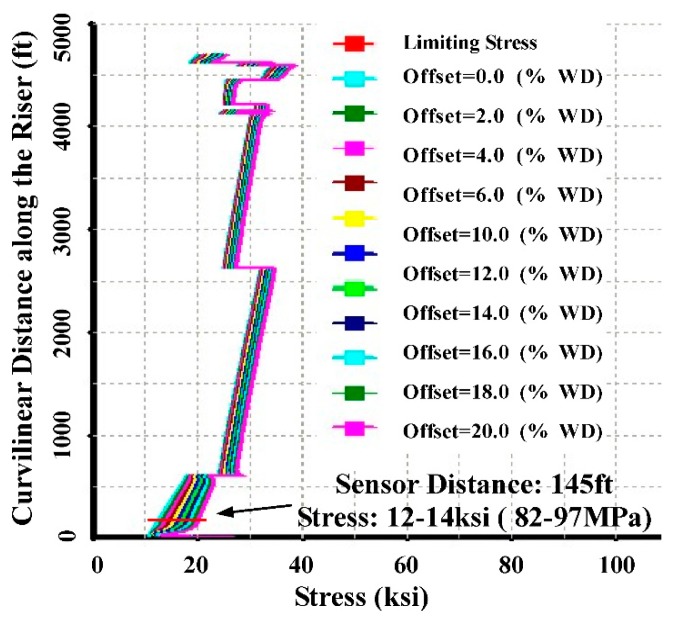
CNOOC mechanical simulation stress results.

The stress results from CNOOC mechanical simulation at different offsets are provided in Figure 6. The abscissa is the stress axis and the ordinate represents the distance to the seabed. When the offset is below 4% water deep (WD), the stress range is 82~95 MPa. The average stress of all measured stresses in drilling and testing conditions is about 86.6 MPa. This value is consistent with the CNOOC mechanical simulation results. 

In summary, the marine test of the stress monitoring sensor system is proved to be successful and its results are reliable, but with the battery power limitations, only one group of FBG wavelengths was uploaded per hour and many other detailed values were lost. Without this limitation, more stress impacts could be found.

## 6. Conclusions

For the monitoring of the stress and bending movements in drilling risers of offshore drilling platforms, a bundle-structured riser stress sensor based on FBG was developed. Thanks to the cooperation with many departments of the CNOOC, a 49-day marine test at water depths of 1365 m and 1252 m was completed on the “HYSY-981” ocean oil drilling platform. Because no welding and pasting are used when the sensor is installed on risers, the installation is convenient, reliable and harmless to risers. The continuous, reasonable and time-consistent data obtained indicates that the sensor worked normally underwater. The monitored results show that the sensor monitors stresses and bending moments accurately. The measured maximum stress is 132.7 MPa and it is below the allowable stress. In drilling & testing conditions, the average riser stress is 86.6 MPa and this value is within the range of CNOOC mechanical simulation results. It is shown that the developed sensor can meet the requirements for monitoring stress in risers under water.
